# Genomic Investigation of the Strawberry Pathogen *Phytophthora fragariae* Indicates Pathogenicity Is Associated With Transcriptional Variation in Three Key Races

**DOI:** 10.3389/fmicb.2020.00490

**Published:** 2020-04-15

**Authors:** Thomas M. Adams, Andrew D. Armitage, Maria K. Sobczyk, Helen J. Bates, Javier F. Tabima, Brent A. Kronmiller, Brett M. Tyler, Niklaus J. Grünwald, Jim M. Dunwell, Charlotte F. Nellist, Richard J. Harrison

**Affiliations:** ^1^Department of Genetics, Genomics and Breeding, NIAB EMR, Kent, United Kingdom; ^2^School of Agriculture, Policy and Development, University of Reading, Reading, United Kingdom; ^3^Department of Botany and Plant Pathology, Center for Genome Biology and Biocomputing, Oregon State University, Corvallis, OR, United States; ^4^Center for Genome Biology and Biocomputing, Oregon State University, Corvallis, OR, United States; ^5^Horticultural Crops Research Unit, Agricultural Research Service, United States Department of Agriculture, Corvallis, OR, United States; ^6^NIAB Cambridge Crop Research, NIAB, Cambridge, United Kingdom

**Keywords:** red core, oomycete, RNA-Seq, host–microbe interactions, nanopore sequencing, population resequencing, race structure

## Abstract

The oomycete *Phytophthora fragariae* is a highly destructive pathogen of cultivated strawberry (*Fragaria* × *ananassa*), causing the root rotting disease, “red core”. The host-pathogen interaction has a well described gene-for-gene resistance relationship, but to date neither candidate avirulence nor resistance genes have been identified. We sequenced a set of American, Canadian, and United Kingdom isolates of known race type, along with three representatives of the closely related pathogen of the raspberry (*Rubus idaeus*), *P. rubi*, and found a clear population structure, with a high degree of nucleotide divergence seen between some race types and abundant private variation associated with race types 4 and 5. In contrast, between isolates defined as United Kingdom races 1, 2, and 3 (UK1-2-3) there was no evidence of gene loss or gain; or the presence of insertions/deletions (INDELs) or Single Nucleotide Polymorphisms (SNPs) within or in proximity to putative pathogenicity genes could be found associated with race variation. Transcriptomic analysis of representative UK1-2-3 isolates revealed abundant expression variation in key effector family genes associated with pathogen race; however, further long read sequencing did not reveal any long range polymorphisms to be associated with avirulence to race UK2 or UK3 resistance, suggesting either control in *trans* or other stable forms of epigenetic modification modulating gene expression. This work reveals the combined power of population resequencing to uncover race structure in pathosystems and *in planta* transcriptomic analysis to identify candidate avirulence genes. This work has implications for the identification of putative avirulence genes in the absence of associated expression data and points toward the need for detailed molecular characterisation of mechanisms of effector regulation and silencing in oomycete plant pathogens.

## Introduction

*Phytophthora fragariae*, the causal agent of red core or red stele root rot, is a highly destructive pathogen of cultivated strawberry (*Fragaria* × *ananassa*), resulting in whole plant collapse. The majority of commercial strawberries grown in the United Kingdom are grown on table tops using soilless substrate, under polytunnels or in glasshouses (Robinson Boyer et al., 2016). *Phytophthora* spp. are a particular problem in these systems due to the ease of spread through the irrigation system via the motile zoospores. Since the first report in Scotland in 1920, this disease has spread to the majority of strawberry growing regions, except China and the Southern Mediterranean regions of Europe (van de Weg, 1997b; Efsa Panel on Plant Health [PLH], 2014). Currently, it is treated as a quarantine pest by the European and Mediterranean Plant Protection Organization (EPPO), where it is listed as an A2 pest (van de Weg, 1997b; EPPO, 2018). The classification of this pathogen has proven controversial, as initially the organism was identified as a single species (Hickman, 1941), but when a *Phytophthora* disease of raspberry (*Rubus idaeus*) was discovered, it was reclassified as *P. fragariae* var. *fragariae* (Wilcox et al., 1993). More recently, the pathogens have been separated into distinct species, *P. fragariae* and *P. rubi*, affecting strawberry and raspberry, respectively. This was supported by sequence analysis of key loci (Man in ’t Veld, 2007), as well as whole genome analyses (Tabima et al., 2018).

It has previously been proposed that the ability of different isolates of *P. fragariae* to cause disease on a variety of *F.* × *ananassa* cultivars can be explained by a gene-for-gene model (van de Weg, 1997a). The model is currently thought to consist of at least eleven resistance genes in *F.* × *ananassa* with eleven corresponding avirulence factors in *P. fragariae* (W. E. van de Weg, Wageningen University and Research, The Netherlands, personal communication). The development of race schemes is country dependent and ones exist for the United Kingdom, United States, and Canada.

All publicly available genome assemblies of *P. fragariae* have to date solely utilised Illumina short read sequencing technologies, resulting in assemblies of 73.68 and 76 Mb, in 1,616 and 8,511 scaffolds, respectively (Gao et al., 2015; Tabima et al., 2017). Recently, long read sequencing technology has been shown to provide assemblies of improved contiguity for *P.* pathogens, specifically the generation of the haplotype-phased assembly of *P. ramorum* (60.5 Mb in 302 primary contigs) using PacBio sequencing (Malar et al., 2019) and the assembly of *P. capsici* (95.2 Mb in 424 scaffolds) using Oxford Nanopore Technology (Cui et al., 2019).

Pathogenomic investigations in *Phytophthora* species of pathosystems with similar gene-for-gene models of resistance have shown a variety of mechanisms through which variation in virulence can be controlled. For instance, in *P. sojae*, the *Avr1d* gene was identified as an RxLR effector recognised by the *Rps1d* resistance gene in soybean (Yin et al., 2013). Studies of the RxLR effector *PiAvr4* from *P. infestans* showed that it was always present in isolates avirulent on potato plants containing the resistance gene *R4*, whereas virulent isolates possessed a frameshift mutation producing a truncated protein (van Poppel et al., 2008). In comparison, *Avr3c* in *P. sojae* was identified in both virulent and avirulent isolates on soybean plants containing *Rps3c*, but in virulent isolates the gene displayed several polymorphisms resulting in a change to the amino acid sequence leading to a failure of recognition by the plant (Dong et al., 2009). Recently, investigations of *P. sojae* and *P. infestans* have identified epigenetic modifications aiding pathogen adaption, with the silencing of relevant effectors leading to the evasion of host immunity (Shan et al., 2004; Qutob et al., 2009, 2013; Dong et al., 2011; Pais et al., 2018).

In this study, we assembled and annotated a population of isolates of *P. fragariae* and the related pathogen of raspberry, *P. rubi*. We identified a subpopulation of *P. fragariae* isolates representing three distinct pathogenicity races (UK1-2-3). This subpopulation was found to be remarkably similar in gene complement, as well as showing little divergence at the nucleotide sequence level. To further investigate the cause of the observed variation in pathogenicity phenotypes, transcriptomic datasets were generated for representative isolates of each pathogenicity race in this subpopulation. This revealed expression level polymorphisms between the isolates, allowing for the generation of candidate lists for *PfAvr1*, *PfAvr2* and *PfAvr3*. A strong candidate for *PfAvr2*, PF003_g27513 was identified as expressed in the BC-16 and A4 (UK2) isolates, yet not expressed in the BC-1 (UK1) and NOV-9 (UK3) isolates. A candidate for *PfAvr3* was also identified, PF009_g26276; it is expressed in NOV-9 (UK3), yet not expressed in BC-1 (UK1), A4 (UK2), and BC-16 (UK2) isolates. Additional sequencing did not reveal long-range polymorphisms influencing the expression of these candidate genes; we therefore suggest control may be in *trans* or due to epigenetic factors.

## Materials and Methods

### Isolate Selection and Sources

A selection of ten isolates of *P. fragariae* were sourced from the Atlantic Food and Horticulture Research Centre (AFHRC), Nova Scotia, Canada. An additional isolate of *P. fragariae*, SCRP245, alongside three *P. rubi* isolates, were sourced from the James Hutton Institute (JHI), Dundee, Scotland (detailed in Table 1).

**TABLE 1:** Summary of *Phytophthora fragariae* and *Phytophthora rubi* isolates used in this study.

Isolate Name and BioSample ID	Species	Location	Date Isolated	Isolated by	Pathogenicity Race
**A4** SAMN07449679	*Phytophthora fragariae*	Unknown	17/12/2001	Unknown	US4 (UK2)
**BC-1** SAMN07449680	*Phytophthora fragariae*	Commercial Strawberry Field, Delta, BC, Canada	05/01/2007	N. L. Nickerson	CA1 (UK1)
**BC-16** SAMN07449681	*Phytophthora fragariae*	Commercial Strawberry Field, Ladner, BC, Canada	05/01/2007	N. L. Nickerson	CA3 (UK2)
**BC-23** SAMN07449682	*Phytophthora fragariae*	Commercial Strawberry Field, Aldergrove, BC, Canada	30/01/2012	N. L. Nickerson	CA5
**NOV-5** SAMN07449684	*Phytophthora fragariae*	Commercial Strawberry Field, Nine Mile River, Hants County, NS, Canada	17/12/2001	N. L. Nickerson	CA1 (UK1)
**NOV-9** SAMN07449687	*Phytophthora fragariae*	Commercial Strawberry Field, Billtown, Kings County, NS, Canada	05/01/2007	N. L. Nickerson	CA2 (UK3)
**NOV-27** SAMN07449683	*Phytophthora fragariae*	Commercial Strawberry Field, Cambridge Station, Kings County, NS, Canada	19/12/2001	N. L. Nickerson	CA2 (UK3)
**NOV-71** SAMN07449685	*Phytophthora fragariae*	Commercial Strawberry Field, Middle Clyde River, Shelburne County, NS, Canada	05/01/2007	N. L. Nickerson	CA2 (UK3)
**NOV-77** SAMN07449686	*Phytophthora fragariae*	Commercial Strawberry Field, Nine Mile River, Hants County, NS, Canada	30/01/2012	N. L. Nickerson	CA5
**ONT-3** SAMN07449688	*Phytophthora fragariae*	Commercial Strawberry Field, Fort Erie, ON, Canada	05/01/2007	N. L. Nickerson	CA4
**SCRP245** SAMN07449689	*Phytophthora fragariae*	Kent, England, United Kingdom	1945	Unknown	Unknown
**SCRP249** SAMN07449690	*Phytophthora rubi*	Germany	1985	Unknown	1
**SCRP324** SAMN07449691	*Phytophthora rubi*	Scotland, United Kingdom	1991	Unknown	1
**SCRP333** SAMN07449692	*Phytophthora rubi*	Scotland, United Kingdom	1985	Unknown	3

### Culturing of Isolates

All work with *P. fragariae* and *P. rubi* was conducted in a Tri-MAT Class-II microbiological safety cabinet. Isolates were routinely subcultured on kidney bean agar (KBA) produced as previously described (Maas, 1972). Isolates were grown by transferring two pieces of between 1 and 4 mm^2^ onto fresh KBA plates, subsequently sealed with Parafilm^®^. Plates were then grown at 20°C in the dark in a Panasonic MIR-254 cooled incubator for between 7 and 14 days.

Mycelia were also grown in liquid pea broth, produced as previously described (Campbell et al., 1989) with the addition of 10 g/L sucrose. These plates were inoculated with five pieces (1 – 4 mm^2^) of mycelium and media, subsequently sealed with Parafilm^®^. These were grown at 20°C for 4 to 5 days in constant darkness.

### Pathogenicity Testing of Isolates

Mother stock plants of *F*. × *ananassa* were maintained in 1 L pots in polytunnels. Runner plants were pinned down into 9 cm diameter pots filled with autoclaved 1:1 peat-based compost:sand. The clones were grown on for 3 weeks to establish their own root system and then were cut from the mother plant. Inoculations were performed as described previously (van de Weg et al., 1996). Plants were placed in a growth chamber with 16/8 h light/dark cycle at a constant 15°C. Inoculated plants stood in a shallow layer of tap water (2 – 7 mm) for the entire experiment and were watered from above twice a week. After 6 weeks, plants were dug up and the roots were rinsed to remove the soil/sand mix. Roots were then assessed for distinctive disease symptoms of “rat’s tails”, which is the dieback from the root tip and “red core”, which is the red discolouration of the internal root visible when longitudinally sliced open. Samples for which infection was unclear were visualised under a light microscope for the presence of oospores. This was performed through squash-mounting of the root tissue, where a sample of root was excised and pressed between a microscope slide and cover slip. This sample was then examined at 40x magnification under high light intensity with a Leitz Dialux 20 light microscope.

### Sequencing of DNA

For Illumina sequencing, gDNA was extracted from 300 mg of freeze-dried mycelium using the Macherey-Nagel NucleoSpin^®^ Plant II Kit. The manufacturer’s protocol was modified by doubling the amount of lysis buffer PL1 used, increasing the incubation following RNase A addition by 5 min, doubling the volume of buffer PC and eluting in two steps with 35 μL of buffer PE warmed to 70°C. For PacBio and Oxford Nanopore Technologies (ONT) sequencing, gDNA was extracted using the Genomic-Tip DNA 100/G extraction kit, following the Tissue Sample method.

To create Illumina PCR-free libraries, DNA was sonicated using a Covaris M220 and size-selected using a BluePippin BDF1510 1.5% gel selecting for 550 bp. Libraries were constructed using the NEBNext enzymes: End repair module (E6050), A-Tailing module (E6053), Blunt T/A ligase (M0367) and Illumina single-indexed adapters. Sequencing was performed to generate 2 × 300 bp reads on a MiSeq^TM^ system using MiSeq Reagent Kit V3 600 cycle (MS-102-3003). PacBio library preparation and sequencing was performed by the Earlham Institute, United Kingdom on a PacBio RS II machine. ONT sequencing libraries were created using the SQK-LSK108 kit following the manufacturer’s protocol and sequenced using the FAH69834 FLO-MIN106 flow cell on a GridION for approximately 28 h.

### Inoculation Time Course Experiment

Growth of isolates for inoculations were performed on fresh KBA plates for approximately 14 days as described above, before the mycelium had reached the plate edge. Plugs of mycelium growing on agar were taken using a flame sterilised 10 mm diameter cork borer and plugs were submerged in chilled stream water. Plates were incubated in constant light for 3 days at 13°C, with the water changed every 24 h. For the final 24 h, chilled Petri’s solution (Judelson et al., 1993) was used. Roots of micropropagated *F.* × *ananassa* “Hapil” plants (GenTech, Dundee, United Kingdom), maintained on *Arabidopsis thaliana* salts (ATS) media (Taylor et al., 2016), were submerged for 1 h before transfering back to ATS plates. These plates were kept at 15°C, with 16/8 h light/dark cycle, with a photosynthetic photon flux (PPF) of 150 μmol m^–2^ s^–1^ provided by fluorescent lamps (FL40SSENW37), in a Panasonic MLR-325H controlled environment chamber. Root tissue was harvested at a selection of time points post-inoculation by rinsing root tissue successively in three beakers of sterile dH_2_O to remove all media. Roots were separated below the crown tissue, flash frozen in liquid nitrogen and stored at −80°C.

Extraction of total RNA from inoculated root tissue was performed similarly to the previously described 3% CTAB_3_ method (Yu et al., 2012). Briefly, plant material was disrupted under liquid nitrogen in a mortar and pestle, previously decontaminated through cleaning with RNaseZap^TM^ solution and baking for 2 h at 230°C to deactivate RNAse enzymes. This material was transferred to a warmed extraction buffer (Yu et al., 2012) containing β-mercaptoethanol with 0.01 g of PVPP added per 0.1 g of frozen tissue. The remaining steps were performed as previously described (Yu et al., 2012), except that 60 μL DEPC-treated H_2_O was used to elute RNA.

Mycelium was grown in liquid pea both and dried on a Q100 90 mm filter paper (Fisher Scientific) using a Büchner funnel and a Büchner flask attached to a vacuum pump. Total RNA was then extracted using the QIAGEN RNEasy Plant Mini kit with the RLC buffer. Extraction was carried out following the manufacturer’s instructions with an additional spin to remove residual ethanol.

RNA was checked for quality using a NanoDrop 1000 spectrophotometer, quantity with a Qubit 2.0 Fluorometer and integrity with a TapeStation 4200 before being prepared for sequencing via Reverse Transcription-Polymerase Chain Reaction (RT-PCR) and sequencing on an Illumina HiSeq^TM^ 4000 by Novogene, Hong Kong, Special Administrative Region, China. Timepoints for sequencing were selected through the detection of β-tubulin transcripts by RT-PCR. Reverse transcription was performed with the SuperScript^TM^ III Reverse Transcriptase kit with an equal amount of RNA used for each sample. The complementary DNA (cDNA) was then analysed by PCR with 200 μM dNTPs, 0.2 μM of each primer (detailed in Supplementary Table S1), 2 μL of cDNA template and 2.5 units of Taq DNA polymerase and the buffer supplied in a 20 μL reaction. Reactions were conducted in a Veriti 96-well thermocycler with an initial denaturation step at 95°C for 30 s, followed by 35 cycles of a denaturation step at 95°C for 30 s, an annealing temperature of 60°C for 30 s and an extension step of 72°C for 30 s. This was followed by a final extension step of 72°C for 5 min and held at 10°C. Products were visualised by gel electrophoresis on a 1% w/v agarose gel at 80 V for 90 min, stained with GelRed. Following this, three biological replicates of samples taken at: 24, 48, and 96 hpi for BC-16, 48 hpi for BC-1 and 72 hpi for NOV-9 were sequenced (Supplementary Figure S1). Additionally, *in vitro* mycelial RNA was sequenced.

*Phytophthora rubi* RNA-Seq reads were sequenced on a HiSeq^TM^ 2000 system.

### Genome Assembly

Prior to assembly, Illumina reads were cleaned and sequencing adaptors were removed with fastq-mcf (Aronesty, 2013). Quality control of PacBio data was performed by the Earlham Institute, Norwich, United Kingdom. ONT reads were basecalled with Albacore version 2.2.7 and adaptors were removed with Porechop version 0.2.0 (Wick, 2018) and the trimmed reads were corrected with Canu version 1.4 (Koren et al., 2017).

Assemblies of isolates sequenced solely with Illumina data were generated with SPAdes version 3.11.0 (Bankevich et al., 2012) with Kmer sizes of 21, 33, 55, 77, 99, and 127. PacBio reads were assembled using FALCON-Unzip (Chin et al., 2016). FALCON version 0.7 + git.7a6ac0d 8e8492c64733a997d72a9359e1275bb57 was used, followed by FALCON-Unzip version 0.4.0 and Quiver (Chin et al., 2013). Error corrected ONT reads were assembled using SMARTdenovo version 1.0.0 (Ruan, 2018).

Following assembly, error correction was performed on ONT assemblies by aligning the reads to the assembly with Minimap2 version 2.8r711-dirty (Li, 2018) to inform ten iterations of Racon version 1.3.1 (Vaser et al., 2017). Following this, reads were again mapped to the assembly and errors were corrected with Nanopolish version 0.9.0 (Simpson, 2018). PacBio and ONT assemblies had Illumina reads mapped with Bowtie 2 version 2.2.6 (Langmead and Salzberg, 2012) and SAMtools version 1.5 (Li et al., 2009) to allow for error correction with ten iterations of Pilon version 1.17 (Walker et al., 2014).

Following assembly, all contigs smaller than 500 bp were discarded and assembly statistics were collected using Quast version 3.0 (Gurevich et al., 2013). BUSCO statistics were collected with BUSCO version 3.0.1 (Simão et al., 2015) using the eukaryota_odb9 database on the assemblies, as the stramenopile database was not available at the time of the analysis. Identification of repetitive sequences was performed with Repeatmasker version open-4.0.5 (Smit et al., 2015) and RepeatModeler version 1.73 (Smit and Hubley, 2015). Transposon related sequences were identified with TransposonPSI release 22nd August 2010 (Haas, 2010).

### Prediction of Gene Models and Effectors

Gene and effector prediction was performed similarly to a previously described method (Armitage et al., 2018). Firstly, raw RNA-Seq reads of BC-16 from both mycelial samples and the inoculation time course were cleaned with fastq-mcf (Aronesty, 2013). Reads from the inoculation time course were first aligned to the *Fragaria vesca* version 1.1 genome (Shulaev et al., 2011) with STAR version 2.5.3a (Dobin et al., 2013) and unmapped reads were kept. These unmapped reads and those from *in vitro* mycelium were mapped to the assembled *P. fragariae* genomes with STAR (Dobin et al., 2013). RNA-Seq data from *P. rubi* were aligned to *P. rubi* assemblies with the same method. Further steps were performed as previously described (Armitage et al., 2018). Additionally, putative apoplastic effectors were identified through the use of ApoplastP (Sperschneider et al., 2018). Statistical significance of the differences between predicted numbers of effector genes was assessed with a Welch two sample *t*-test in R version 3.4.3 (R Core Team, 2017). Supporting annotation data for UK2 isolate BC-16 (Supplementary Table S2).

### Gene Orthology Analysis

Orthologue identification was performed using OrthoFinder version 1.1.10 (Emms and Kelly, 2015) on predicted proteins from all sequenced *P. fragariae* and *P. rubi* isolates (Supplementary Table S3). Orthogroups were investigated for expanded and unique groups for races UK1, UK2 and UK3. Unique orthogroups were those containing proteins from only one race and expanded orthogroups were those containing more proteins from a specific race than other races. Venn diagrams were created with the VennDiagram R package version 1.6.20 (Chen and Boutros, 2011) in R version 3.2.5 (R Core Team, 2016).

### Identification and Analysis of Variant Sites and Population Structure

Variant sites were identified through the use of the GATK version 3.6 HaplotypeCaller in diploid mode (McKenna et al., 2010) following alignment of Illumina reads for all sequenced isolates to the FALCON-Unzip assembled genome with Bowtie 2 version 2.2.6 (Langmead and Salzberg, 2012) and filtered with vcflib (Garrison, 2012) and VCFTools (Danecek et al., 2011). Additionally, structural variants were identified with SvABA (Wala et al., 2018) following the alignment of Illumina reads from all sequenced isolates to the FALCON-Unzip assembled genome with BWA-mem version 0.7.15 (Li, 2013). Population structure was assessed with fastSTRUCTURE (Raj et al., 2014) following conversion of the input file with Plink version 1.90 beta (Purcell et al., 2007). Finally, a custom Python script was used to identify variant sites private to races UK1, UK2, or UK3 (see Availability of Computer Code below).

### Assessment of Gene Expression Levels and the Identification of Candidate Avirulence Genes

RNA-Seq reads of BC-1, BC-16, and NOV-9 were aligned to the assembled genomes of BC-1, BC-16, and NOV-9 as described above. Expression levels and differentially expressed genes were identified with featureCounts version 1.5.2 (Liao et al., 2014) and the DESeq2 version 1.10.1 R package (Love et al., 2014). Multiple test correction was performed as part of the analysis within the DESeq2 package.

Candidate avirulence genes were identified through the analysis of uniquely expressed genes and uniquely differentially expressed genes for each of the three isolates. Uniquely expressed genes were those with a Fragments Per Kilobase of transcript per Million mapped reads (FPKM) value greater than or equal to 5 in any time point from the inoculation time course experiment for a single isolate. Uniquely differentially expressed genes were those with a minimum LFC of 3 and a *p*-Value threshold of 0.05. Genes were compared between isolates through the use of orthology group assignments described above and scored on a one to six scale, with five and six deemed high confidence and one and two deemed as low confidence.

To identify homologous genes in other *Phytophthora* spp., candidate RxLRs were processed by SignalP-5.0 (Armenteros et al., 2019) to identify the cleavage site. The signal peptide sequence was removed before being submitted for a tblastn (Altschul et al., 1997) search on GenBank; interesting top hits are reported.

The expression levels of a strong candidate *PfAvr2* gene, PF003_g27513 and a candidate *PfAvr3* gene, PF009_g26276, were assessed via RT-qPCR. RNA-Seq results for reference genes identified previously in *P. parasitica* (Yan and Liou, 2006) were examined for stability of expression levels, resulting in the selection of β-tubulin (PF003_g4288) and WS41 (PF003_g28439) as reference genes. The gDNA of P*seudomonas syringae* pv. *maculicola* 5422 and primers for 16S (Clifford et al., 2012) were used as an inter-plate calibrator. Primers were designed using the modified Primer3 version 2.3.7 implemented in Geneious R10 (Untergasser et al., 2012). Reverse transcription was performed on three biological replicates of each sequenced timepoint, alongside: 24 hpi, 72 hpi and 96 hpi for BC-1 and 24 hpi, 48 hpi and 96 hpi for NOV-9 with the QuantiTect Reverse Transcription Kit. Quantitative PCR (qPCR) was then performed in a CFX96^TM^ Real-Time PCR detection system in 10 μL reactions of: 5 μL of 2× qPCRBIO SyGreen Mix Lo-Rox, 2 μL of a 1:3 dilution of the cDNA sample in dH_2_O and 0.4 μL of each 10 μM primer and 2.2 μL dH_2_O. The reaction was run with the following conditions: 95°C for 3 min, 39 cycles of 95°C for 10 s, 62°C for 10 s and 72°C for 30 s. This was followed by 95°C for 10 s, and a 5 s step ranging from 65°C to 95°C by 0.5°C every cycle. At least two technical replicates for each sample were performed and the melt curve results were analysed to ensure the correct product was detected. Relative gene expression was calculated using the comparative cycle threshold (C_T_) method (Livak and Schmittgen, 2001). Where there was a difference of at least 1 C_T_ value between the minimum and maximum results for technical replicates, further reactions were conducted. Outlier technical replicates were first identified as being outside 1.5 times the interquartile range. Following this, additional outliers were identified using the Grubb’s test and excluded from the analysis. Technical replicates were averaged for each biological replicate and expression values were calculated as the mean of the three biological replicates.

### Investigation of *cis* and *trans* Variations for Strong Candidate Avirulence Gene

The regions upstream and downstream of PF003_g27513 and PF009_g26276 in the FALCON-Unzip assembly of BC-16 and the SMART *de novo* assembly of NOV-9 were aligned with MAFFT in Geneious R10 (Katoh et al., 2002; Katoh and Standley, 2013). Variant sites were identified through visual inspection of the alignment alongside visualisation of aligned short reads from BC-16 and NOV-9 to both assemblies with Bowtie 2 version 2.2.6 (Langmead and Salzberg, 2012) in IGV (Thorvaldsdóttir et al., 2013).

Transcription factors were identified with a previously described HMM of transcription factors and transcriptional regulators in Stramenopiles (Buitrago-Flórez et al., 2014). These proteins were then analysed through methods described above for gene loss or gain, nearby variant sites and differential expression between isolates.

### Availability of Computer Code

All computer code is available at: https://github.com/harri- sonlab/phytophthora_fragariae, https://github.com/harrisonlab/phytophthora_rubi, and https://github.com/harrisonlab/popgen/blob/master/snp/vcf_find_difference_pop.py.

## Results

### Race Typing Allowed Standardisation of United States and United Kingdom Race Nomenclature

**FIGURE 1:**
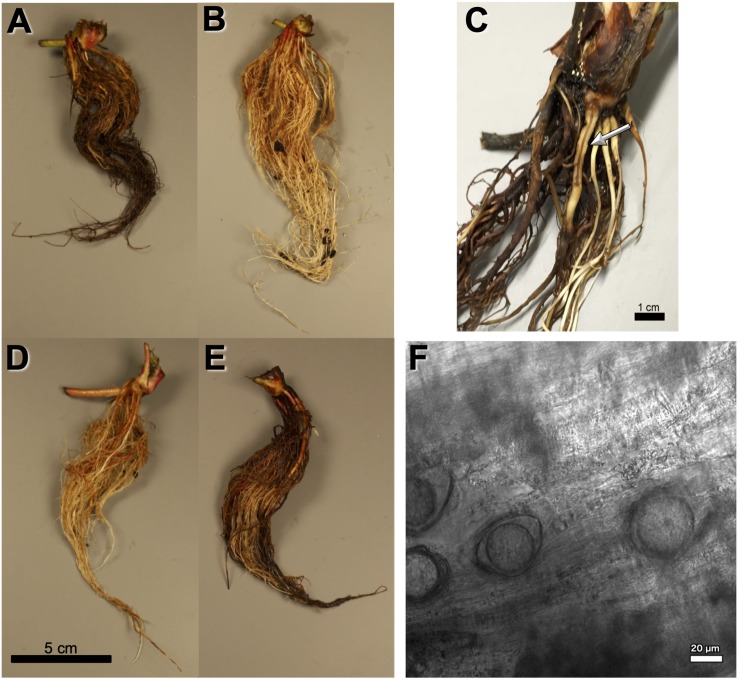
Observed *Phytophthora fragariae* symptoms in the cultivated strawberry (*Fragaria* × *ananassa*). **(A,B,D,E)** Roots of *Fragaria* × *ananassa* harvested 6 weeks after inoculation with *Phytophthora fragariae* mycelial slurry. **(A)** Successful infection of the *P. fragariae* isolate NOV-27 (race CA2) on a susceptible “Redgauntlet” plant (*Rpf2* only). **(B)** Unsuccessful infection of the *P. fragariae* isolate A4 (US4/UK2) on a resistant “Redgauntlet” plant (*Rpf2* only). **(C)** Example of “red core” symptoms observed in “Hapil” roots infected with BC-16, 3 weeks post-inoculation. **(D)** Unsuccessful infection of the *P. fragariae* isolate NOV-27 (race CA2) on a resistant “Allstar” plant (*Rpf1*, *Rpf2* and *Rpf3*). **(E)** Successful infection of the *P. fragariae* isolate A4 (race US4/UK2) on a susceptible “Hapil” plant (no *Rpf* genes). **(F)** Example of BC-16 oospores observed in “Hapil” roots, 3 weeks post-inoculation, confirming infection.

The *P. fragariae* isolates A4 (race US4), BC-1 (race CA1), BC-16 (race CA3), NOV-5 (race CA1), NOV-9 (race CA2), NOV-27 (race CA2), and NOV-71 (race CA2) (Table 1) were phenotyped on a differential series of four *F.* × *ananassa* cultivars with known resistance. These were: “Allstar” (containing *Rpf1*, *Rpf2*, and *Rpf3*), “Cambridge Vigour” (containing *Rpf2* and *Rpf3*), “Hapil” (containing no resistance genes), and “Redgauntlet” (containing *Rpf2*) (van de Weg et al., 1996; R. Harrison, unpublished). Following assessment of below ground symptoms, it was shown that race CA1 was equivalent to UK1, race CA2 was equivalent to UK3, race CA3 was equivalent to UK2 and race US4 was equivalent to UK2 (Figure 1 and Table 2).

**TABLE 2:** *Phytophthora fragariae* race structure detected by three differential strawberry (*Fragaria* × *ananassa*) accessions and categorisation into UK race scheme.

		*Phytophthora fragariae* Isolate^b^						
		
Cultivar^a^	Mock	BC-1	BC-16	NOV-9	A4	NOV-27	NOV-5	NOV-71
“Allstar”_1,__2,__3_	0^4/4^	0^8/8^	0^7/7^	0^4/4^	0^3/4^	0^2/3^	0^3/3^	0^4/5^
“Cambridge Vigour”_2,__3_	0^5/5^	+ ^7/9^	0^5/5^	0^8/8^	0^5/5^	0^4/5^	+ ^5/5^	0^4/5^
“Hapil”	0^12/12^	+ ^10/10^	+^10/10^	+ ^10/10^	+ ^4/5^	+ ^5/5^	+ ^3/5^	+ ^5/5^
“Redgauntlet”_2_	0^12/12^	+ ^7/10^	0^9/10^	+ ^9/10^	0^5/5^	+ ^5/5^	+ ^5/5^	+ ^5/5^
Deduced UK race	N/A	Race 1	Race 2	Race 3	Race 2	Race 3	Race 1	Race 3

*^*a*^Known resistance genes in the *Fragaria* × *ananassa* cultivars. ^*b*^Number of replicates showing the recorded phenotype compared to the number of replicates tested; “ + ” representing a successful infection and “0” representing resistance.*

### A Highly Contiguous Genome Assembly of BC-16

Long read PacBio sequencing generated a highly contiguous *P. fragariae* isolate BC-16 (UK2) reference genome of 91 Mb in 180 contigs with an N50 value of 923.5 kb (Table 3). This was slightly larger than the closely related, well studied species from Clade 7, *P. sojae*, at 83 Mb (Table 3; Tyler et al., 2006). The BC-16 assembly contained 266 of 303 eukaryotic Benchmarking Universal Single-Copy Orthologs (BUSCO) genes (Table 4), compared to 270 in *P. sojae* (Armitage et al., 2018) and so likely represented a similar completeness of the genome as the *P. sojae* assembly. The *P. fragariae* genome was shown to be highly repeat rich, with 38% of the assembly identified as repetitive or low complexity, a larger value than the 29% shown for *P. sojae* (Armitage et al., 2018). A total of 37,049 genes encoding 37,346 proteins were predicted in the BC-16 assembly, consisting of 20,222 genes predicted by BRAKER1 (Hoff et al., 2016) and 17,131 additional genes added from CodingQuarry (Testa et al., 2015). From these gene models, 486 putative RxLR effectors, 82 putative crinkler effectors (CRNs) and 1,274 putative apoplastic effectors were identified (Table 5). Additionally, a total of 4,054 low confidence gene models were added from intergenic ORFs identified as putative effectors. These consisted of 566 putative RxLRs, 5 putative CRNs and 3,483 putative apoplastic effectors. This resulted in a combined total of 41,103 genes encoding 41,400 proteins with 1,052 putative RxLRs, 85 putative CRNs, and 4,757 putative apoplastic effectors (Tables 3, 5).

**TABLE 3:** Long read PacBio sequencing generated a highly contiguous *Phytophthora fragariae* BC-16 (UK2) reference genome.

	*Phytophthora fragariae*	*Phytophthora sojae*

	BC-16	P6497
Assembly size (Mb)	90.97	82.60
Number of contigs	180	862
N50 (kb)	923.5	386.0
L50	33	61
Repeatmasked	38%	29%
Genes	37,049 (41,103)	26,584
RxLRs	486 (1,052)	350
CRNs	82 (85)	40
Apoplastic effectors	1,274 (4,757)	N/A

*Assembly and annotation statistics compared to *Phytophthora sojae* P6497 (Tyler et al., 2006). Values in brackets include low confidence gene models from open reading frames.*

**TABLE 4:** Comparable assembly statistics and gene predictions in resequenced isolates of *Phytophthora fragariae* and *Phytophthora rubi*.

		*Phytophthora fragariae*										*Phytophthora rubi*		
			
	A4	BC-1	BC-16	BC-23	NOV-5	NOV-9	NOV-27	NOV-71	NOV-77	ONT-3	SCRP245	SCRP249	SCRP324	SCRP333
Assembly Size (Mb)	79.08	79.10	90.97	78.26	78.99	79.43	79.75	78.37	78.80	79.06	77.84	77.79	77.91	77.82
Contig Number (≧ 500 bp)	13,446	11,556	180	13,191	13,531	11,801	12,489	12,212	13,321	13,265	13,231	14,023	13,946	13,679
N50 (kb)	18.2	21.8	923.5	18.2	17.9	21.5	19.4	20.2	18.9	18.8	18.2	16.6	16.9	16.9
L50	1,116	953	33	1,119	1,134	978	1,046	1,016	1,101	1,104	1,120	1,232	1,218	1,210
Repeatmasked	31%	31%	38%	31%	31%	31%	31%	31%	31%	31%	31%	31%	31%	30%
Genes^a^	30,180 (33,623)	30,375 (33,691)	37,049 (41,103)	29,960 (33,143)	30,248 (33,699)	29,986 (33,527)	30,600 (33,797)	29,708 (33,143)	30,099 (33,580)	30,180 (33,415)	30,202 (33,223)	31,458 (34,139)	30,235 (33,263)	32,623 (35,223)
Single-Copy BUSCO genes	274 (90%)	274 (90%)	266 (88%)	275 (91%)	273 (90%)	273 (90%)	273 (90%)	274 (90%)	272 (90%)	277 (91%)	273 (90%)	273 (90%)	274 (90%)	275 (91%)
Duplicated BUSCO genes	6 (2%)	6 (2%)	9 (3%)	5 (2%)	7 (2%)	6 (2%)	6 (2%)	6 (2%)	8 (2.5%)	7 (2%)	7 (2.5%)	8 (3%)	8 (3%)	7 (2%)
Fragmented BUSCO genes	6 (2%)	6 (2%)	5 (1.5%)	7 (2%)	6 (2%)	7 (2%)	7 (2%)	6 (2%)	6 (2%)	4 (1%)	7 (2.5%)	3 (1%)	3 (1%)	3 (1%)
Missing BUSCO genes	17 (6%)	17 (6%)	23 (7.5%)	16 (5%)	17 (6%)	17 (6%)	17 (6%)	17 (6%)	17 (5.5%)	15 (5%)	16 (5%)	19 (6%)	18 (6%)	18 (6%)

*^*a*^Values in brackets include low confidence gene models from open reading frames.*

**TABLE 5:** Effector gene predictions in resequenced isolates of *Phytophthora fragariae* and *Phytophthora rubi*.

		*Phytophthora fragariae*										*Phytophthora rubi*		
			
	A4	BC-1	BC-16	BC-23	NOV-5	NOV-9	NOV-27	NOV-71	NOV-77	ONT-3	SCRP245	SCRP249	SCRP324	SCRP333
Secreted proteins^a^	3,637	3,601	4,217	3,611	3,626	3,637	3,690	3,633	3,620	3,658	3,581	3,697	3,683	3,832
RxLR HMM^b^	194	194	218	188	196	186	183	191	166	191	196	197	195	207
RxLR-EER Regex^c^	178	184	208	176	186	174	172	182	150	178	185	188	176	188
RxLR Regex	371	367	445	364	370	356	369	374	341	367	370	363	350	359
Final RxLR effectors^*d*^	410 (935)	405 (919)	486 (1,052)	402 (928)	408 (918)	397 (951)	405 (899)	412 (931)	378 (934)	403 (898)	407 (882)	407 (882)	395 (861)	410 (826)
CRN LFLAK HMM^c^	85	83	114	86	84	82	87	90	86	90	90	156	93	154
CRN DWL HMM^c^	95	87	121	96	91	96	93	101	83	90	87	139	102	142
Final CRNs^d,e^	55 (68)	53 (59)	82 (87)	55 (62)	53 (67)	50 (61)	57 (63)	59 (71)	62 (75)	61 (74)	60 (68)	128 (132)	71 (85)	128 (134)
Apoplastic effectors^d,f^	991 (3,896)	1,002 (3,798)	1,274 (4,757)	980 (3,630)	986 (3,913)	1,007 (3,983)	1,011 (3,708)	1,007 (3,911)	1,010 (3,922)	982 (3,709)	984 (3,522)	1,017 (3,266)	1,059 (3,607)	1,090 (3,268)

*^*a*^SignalP: Nielsen et al., 1997; Bendtsen et al., 2004; Petersen et al., 2011 and Phobius: Käll et al., 2004. ^*b*^Whisson et al. (2007). ^*c*^Armitage et al. (2018). ^*d*^Values in brackets include low confidence gene models from open reading frames. ^*e*^Both LFLAK and DWL HMM models present (Armitage et al., 2018). ^*f*^ApoplastP: Sperschneider et al. (2018).*

### A Comparable Number of Effector Genes Were Identified Through Resequencing of Isolates of *Phytophthora fragariae* and *Phytophthora rubi*

Ten isolates of *P. fragariae* and three isolates of *P. rubi* were additionally resequenced, *de novo* assembled and annotated (Tables 1, 4). These isolates showed similar assembly statistics within and between species; an average of 79 Mb in 12,804 contigs with an N50 of 19.3 kb in *P. fragariae*, compared to an average of 78 Mb in 13,882 contigs with an N50 of 16.8 kb in *P. rubi*. All assemblies showed 31% of the assembly was identified as repetitive or low complexity sequences. On average both *P. fragariae* and *P. rubi* showed high levels of completeness, with an average of 274/303 BUSCO genes identified as single copies in these assemblies. Interestingly, a total of 17 genes were consistently not identified in Illumina, PacBio and Nanopore assemblies of *P. fragariae* and *P. rubi* isolates, suggesting these genes may be absent from these species and as such may not be considered true eukaryotic BUSCO genes. An average of 67 CRNs were predicted in *P. fragariae* and an average of 118 CRNs in *P. rubi*. A significantly (*p* = 0.013) smaller number of RxLRs were predicted in *P. rubi* isolates than *P. fragariae* isolates alongside a significantly (*p* = 0.039) smaller number of apoplastic effectors predicted in *P. rubi* than *P. fragariae* (Table 5). However, it is important to note that these effectors were predicted from a subset of secreted proteins, of which there were significantly (*p* = 0.030) fewer predicted in *P. rubi* than in *P. fragariae* (Table 5).

### No Distinguishing Gene Loss or Gain; Or the Presence of INDELs or SNPs in Candidate Avirulence Genes Could Be Found Associated With Race Variation

**FIGURE 2:**
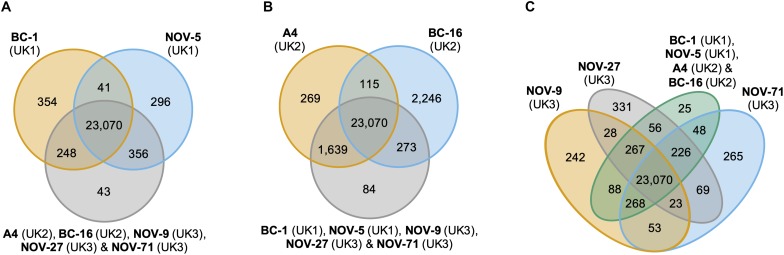
Analysis of unique and expanded orthogroups for *Phytophthora fragariae* isolates of the UK1, UK2, and UK3 races did not lead to the identification of candidate avirulence genes. Orthology groups were identified by OrthoFinder (Emms and Kelly, 2015) and Venn diagrams were plotted using the VennDiagram R package (Chen and Boutros, 2011) in R Core Team (2016). **(A)** Analysis focused on the *P. fragariae* isolates of race UK1: BC-1 and NOV-5 compared to isolates of races UK2 and UK3. **(B)** Analysis focused on the *P. fragariae* isolates of race UK2: A4 and BC-16 compared to isolates of races UK1 and UK3. **(C)** Analysis focused on the *P. fragariae* isolates of race UK3: NOV-5, NOV-27 and NOV-71 compared to isolates of races UK1 and UK2.

An orthology analysis of all predicted proteins from the isolates of *P. fragariae* and *P. rubi* assigned 481,942 (98.7%) proteins to 38,891 orthogroups. Of these groups, 17,101 contained at least one protein from all fourteen sequenced isolates and 13,132 of these groups consisted entirely of single-copy proteins from each isolate. There were 2,345 of these groups unique to *P. rubi* and 1,911 of these groups unique to *P. fragariae*. Analysis of unique and expanded orthogroups for isolates of the UK1, UK2 and UK3 races did not lead to the identification of candidate avirulence genes, as these groups did not contain putative effector genes (Figure 2).

**FIGURE 3:**
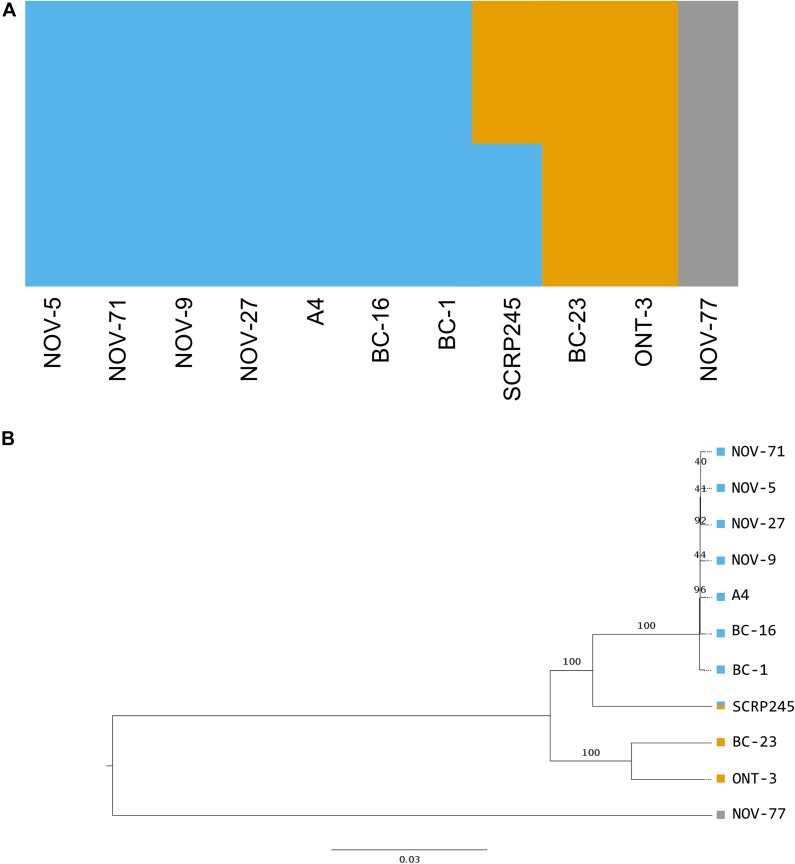
Phylogenetic and population analysis of high quality, biallelic SNP sites split the isolates into three populations with SCRP245, potentially forming an ancestral or hybrid isolate between the UK1-2-3 population and the population represented by BC-23 and ONT-3. **(A)** Distruct plot of fastSTRUCTURE (Raj et al., 2014) results carried out on all sequenced isolates of *Phytophthora fragariae*. Each colour represents a different population. 1,469 variant sites were retained for this analysis after filtering. **(B)** Neighbour joining tree based on 545,365 high quality, biallelic SNP sites, node labels represent the number of bootstrap replicates supporting the node. Variant sites were identified by aligning Illumina reads of all the sequenced isolates to the reference assembly of the BC-16 isolate of *P. fragariae* with Bowtie 2 (Langmead and Salzberg, 2012) and analysis with the Genome Analysis Toolkit (GATK) haplotypecaller (McKenna et al., 2010). Sites were filtered with VCFtools (Danecek et al., 2011) and VCFlib (Garrison, 2012) to leave only high quality, biallelic SNP sites.

A total of 725,444 SNP sites and 95,478 small INDELs were identified within the *P. fragariae* and *P. rubi* isolates by the Genome Analysis ToolKit (GATK) haplotypecaller (McKenna et al., 2010) and 80,388 indels and 7,020 structural variants were identified by SvABA (Wala et al., 2018). Analysis of high quality, biallelic SNP sites allowed the identification of a distinct population consisting of the isolates of race UK1, UK2 and UK3, hereafter referred to as the UK1-2-3 population, with SCRP245, the only United Kingdom isolate, potentially forming an ancestral or hybrid isolate between the UK1-2-3 population and the population represented by BC-23 and ONT-3 (Figure 3). Additionally, clear separation between isolates of *P. fragariae* and *P. rubi* was observed. Further analysis within the UK1-2-3 population allowed for the identification of private variants, which were only present in isolates of one of the three races in this population. This resulted in the identification of twelve private variants in race UK2, shared between both A4 and BC-16; however, neither the genes they fell within or were neighbours to, were predicted to encode effectors or secreted proteins and likely did not explain the differences in pathogenicity (Supplementary Table S4).

### Wide Scale Transcriptional Reprogramming of Effectors During Strawberry Infection

**FIGURE 4:**
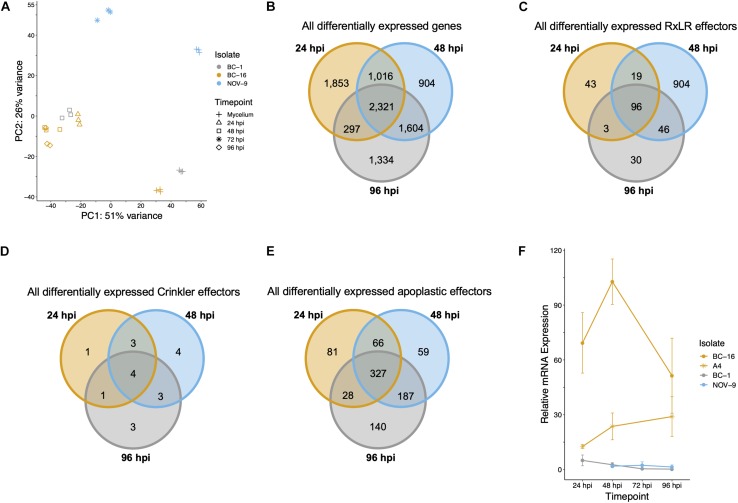
Analysis of expression data showed grouping of biological replicates, a separation of the BC-16 timepoints, changes during the infection process by the BC-16 isolate and confirmation of the differential expression of a candidate avirulence gene. **(A)** Principal component analysis of the differentially expressed transcripts for all analysed RNA-Seq experiments. RNA-Seq reads were aligned to the assembly of the BC-16 isolate of *Phytophthora fragariae* using STAR version 2.5.3a (Dobin et al., 2013). Predicted transcripts were then quantified with featureCounts version 1.5.2 (Liao et al., 2014) and differential expression was identified with the DESeq2 version 1.10.1 R package (Love et al., 2014). Following this, an rlog transformation of the expression data was plotted as a principal component analysis with R Core Team (2016). **(B)** Venn diagram of all differentially expressed transcripts during the BC-16 infection timecourse experiment. **(C)** Venn diagram of all differentially expressed predicted RxLR effectors during the BC-16 infection timecourse experiment. **(D)** Venn diagram of all differentially expressed predicted Crinkler effectors during the BC-16 infection timecourse experiment. **(E)** Venn diagram of all differentially expressed predicted apoplastic effectors during the BC-16 infection timecourse experiment. Venn diagrams were plotted using the VennDiagram R package (Chen and Boutros, 2011) in R Core Team (2016). **(F)** Quantitative reverse transcription PCR of a strong candidate for the avirulence gene possessed by BC-16 and A4, but not BC-1 and NOV-9 (PF003_g27513.t1). Plots created by the ggplot2 R package (Wickham, 2016) in R version 3.4.3 (R Core Team, 2017).

RNA-Seq data were generated from an inoculation time course experiment for representatives of races UK1, UK2 and UK3; BC-1, BC-16 and NOV-9, respectively. Following determination of expression levels of the predicted genes in both *in planta* and mycelia samples, a correlation analysis showed biological replicates of each timepoint grouped together (Figure 4A). Additionally, the 48 h post-inoculation (hpi) BC-1 time point grouped with the BC-16 24 hpi time point, suggesting these may represent similar points in the infection process. However, clear separation between the BC-16 timepoints was observed (Figure 4A). A total of 13,240 transcripts from BC-16 (32%) showed expression above a FPKM value threshold of five in at least one sequenced BC-16 time point and 9,329 (23%) of these showed evidence of differential expression in at least one sequenced *in planta* time point compared to mycelium grown in artificial media, suggesting large scale transcriptional reprogramming. As three time points post-inoculation were sequenced for BC-16, the changes in expression over the course of the infection process was investigated. A total of 2,321 transcripts (6%) showed a Log_2_ Fold Change (LFC) greater than or equal to one or less than or equal to minus one, representing a general reprofiling during infection in comparison to growth in artificial media. Of transcripts differentially expressed *in planta* compared to artificial media, fewer transcripts were differentially expressed at both 24 hpi and 96 hpi than between sequential timepoints (297 compared to 1,016 and 1,604 transcripts). This suggested that changes during the progress of infection were captured by this dataset (Figure 4B).

Levels of expression of effector genes were also assessed. A total of 274 (26%) RxLR effectors, 27 (31%) CRNs and 880 (18%) putative apoplastic effectors from BC-16 showed evidence of expression above the FPKM threshold of 5 in at least one sequenced time point. The majority of these effector genes also showed evidence of differential expression during *in planta* time points compared to *in vitro* mycelium. A total of 253 (24%) RxLR effectors, 19 (22%) CRNs and 888 (18%) putative apoplastic effectors showed an LFC greater than or equal to one or less than or equal to minus one, representing wide scale transcriptional reprogramming of effector genes during infection (Figures 4C–E). Ranking of the fifty highest expressed genes *in planta* with a LFC of ≥3 in comparison to its respective mycelium, identified four putative RxLR genes that were upregulated by all three isolates (Table 6). These genes represent putative core *P. fragariae* RxLR’s important for pathogenicity on strawberry. Interestingly, a BLASTP search of one of these putative core effectors, PF003_g16448 (amino acids 19-139), showed homology to *P. sojae Avr1b* (58% pairwise identity), GenBank accession AF449625 (Shan et al., 2004).

**TABLE 6:** Conserved putative core *Phytophthora fragariae* RxLR effector candidates important for pathogenicity on strawberry (*Fragaria* × *ananassa*) from isolates BC-1, BC-16 and NOV-9.

		Fragments Per Kilobase of transcript per Million mapped reads (FPKM)								
			
		BC-1 (UK1)			BC-16 (UK2)			NOV-9 (UK3)		
					
Orthogroup	BC-16 gene ID	Mycelium	48 hpi	Mycelium	24 hpi	48 hpi	96 hpi	Mycelium	72 hpi	Avr homology
OG0010423	PF003_g35418	94	5,354	192	6,578	4,986	2,961	12	3,868	
OG0018019	PF003_g6480	241	3,544	235	2,113	1,977	1,717	231	2,501	
OG0011458	PF003_g16448	4	2,899	4	4,656	2,108	1,090	7	1,699	*P. sojae Avr1b*^a^
OG0021012	PF003_g16234	36	1,345	31	1,181	827	674	23	737	

*Conserved RxLR candidates in the 50 highest expressed genes *in planta* with a log fold change (LFC) of ≥3, based on comparison to respective mycelium. The BC-16 24 h post-inoculation (hpi) timepoint was used for BC-16. ^*a*^Top hit from GenBank tblastn search is AF449623, *Phytophthora sojae Avr1b* (Shan et al., 2004).*

### RxLR Effector PF003_g27513 Is a Strong Candidate for *PfAvr2*

Comparing transcripts with the highest LFC (*in planta* vs. mycelium) between isolates led to the identification of the putative RxLR effector encoding transcript PF003_g27513.t1 as a potential candidate for *PfAvr2* in BC-16. PF003_g27513 had a peak FPKM value in BC-16 of 9,392 compared to peaks of 0 and 33, in BC-1 and NOV-9, respectively (Supplementary Table S5). Subsequent RT-qPCR analysis of further timepoints in the *in planta* timecourse supported the findings of the RNA-Seq timepoints and showed the expression of putative *PfAvr2* in the UK2 isolates BC-16 and A4 was significantly (*p* < 0.05) different to those from all samples of BC-1 (UK1) and NOV-9 (UK3; Figure 4F). A BLASTP search of PF003_g16448 (amino acids 26-137) revealed homology to *P. sojae Avh6/Avr1d* (37% pairwise identity), GenBank accession JN253642 (Wang et al., 2011; Na et al., 2013).

**FIGURE 5:**
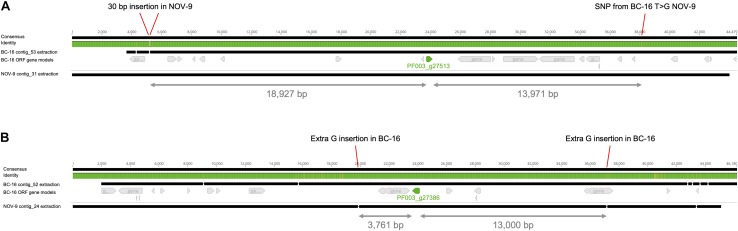
Differential expression of putative *PfAvr2* and *PfAvr3* is not due to sequence variation in *Phytophthora fragariae* BC-16 (UK2) and NOV-9 (UK3) genomes. Regions surrounding candidate avirulence genes, *PfAvr2* and *PfAvr3*, from BC-16 and NOV-9 aligned with MAFFT in Geneious R10 (Katoh et al., 2002; Katoh and Standley, 2013). Black bars indicate contiguous sequences and gaps are represented by dashes. Identity is shown for all sequences in the alignment, green denotes residues at that position are the same across all sequences, yellow denotes less than complete identity and red denotes very low identity for the given position. **(A)** Putative *PfAvr2*, showing a 30 bp insertion in the NOV-9 sequence upstream of PF003_g27513 (shown in green) and a T to G SNP in NOV-9 downstream of the gene of interest. **(B)** Putative *PfAvr3*, showing an extra G insertion in BC-16 upstream of PF003_g27386 (an orthologue of PF009_g26267; shown in green) and an extra G insertion in BC-16 3,761 bp downstream.

The surrounding sequence of putative *PfAvr2* in BC-16, BC-1 and NOV-9 was investigated for sequence variants that may explain the expression difference. As no variants were identified near the gene from the above mentioned variant panel, an assembly of the NOV-9 isolate was created from Nanopore sequencing data, producing an assembly of 93.72 Mbp in 124 contigs with an N50 of 1,260 Kb. This resulted in the identification of a SNP from T in BC-16 to G in NOV-9 ∼14 Kb downstream of the stop codon and a 30 bp insertion in NOV-9 ∼19 Kb upstream of the start codon (Figure 5A). The upstream variant appeared to be a sequencing error following the investigation of the alignment of short reads of BC-16 and NOV-9 to each assembly and so was rejected. Expression of the genes surrounding PF003_g27513 was investigated and PF003_g27514, directly upstream of putative *PfAvr2* is expressed by all three isolates (Supplementary Table S5).

Additionally, putative transcription factors and transcriptional regulators were identified in all sequenced genomes. This resulted in the identification of 269 genes in the BC-16 isolate of *P. fragariae*. However, analysis of gene loss or gain and an investigation of variant sites showed no race specific differences, though expression level variation was observed.

### RxLR Effector PF009_g26276 Is a Putative Candidate for *PfAvr3*

Further analysis of the RNA-Seq datasets identified the putative RxLR effector encoding transcript PF009_g26276 (an orthologue of PF003_g27386) as a potential candidate for *PfAvr3* in NOV-9, with a peak FPKM value in NOV-9 of 199 compared to *in planta* peaks of 6 and 12 in BC-1 and BC-16, respectively (Supplementary Table S6). Similar to putative *PfAvr2*, no sequence differences in putative *PfAvr3* were observed between the three isolates. Analysis of the surrounding region between NOV-9 and BC-16 showed no sequence differences 13,000 bp upstream and 3,761 bp downstream of PF009_g26276 (Figure 5B). The two genes upstream from putative *PfAvr3* are not expressed in any isolate (Supplementary Table S6), whereas the gene directly downstream in BC-16, PF003_g27385, is expressed by all the three isolates analysed.

Subsequent RT-qPCR analysis of further timepoints in the *in planta* time course revealed that the putative *PfAvr3* was not expressed during any timepoints by the UK1 isolate BC-1 or by the UK2 isolates BC-16 and A4 (Supplementary Figure S2). However, absolute expression of PF009_g26276 in NOV-9 remained low. Expression in NOV-9 at 72 hpi was significantly (*p* < 0.05) greater than those from all samples of BC-1, BC-16, and A4.

### Candidate Genes for *PfAvr1* Identified From Expression Level Variation

Analysis of the RNA-Seq datasets did not reveal an obvious candidate for *PfAvr1*. Further analysis using custom scripts identified genes, which were uniquely expressed in BC-1 and those uniquely differentially expressed. These genes were scored for confidence of race avirulence determinant; high, medium and low. No genes were identified in the high confidence class in BC-1, but four genes were scored as medium confidence, one of which was a putative apoplastic effector (Supplementary Table S7). The analysis was repeated for BC-16 and NOV-9, in case the putative RxLR candidates are not *PfAvr*2 and *PfAvr3*, respectively (Supplementary Table S7).

## Discussion

Understanding pathogenicity of plant pathogens is critical for developing durable resistance strategies. *P. fragariae* is a continuing threat to strawberry production. The UK1-2-3 population displayed clear separation from the other isolates of *P. fragariae* in this study. Two putative avirulence candidates for UK2 and UK3 were identified through population resequencing and analyses of gene expression during *P. fragariae* infection. We have shown that there are no distinguishing gene loss or gain events, INDELs or SNPs associated with race variation in UK1-2-3. Our results also suggest that the polymorphisms associated with avirulence to race UK2 and UK3 resistance is controlled *in trans* or with other stable forms of epigenetic modulating gene expression.

This study utilised long read sequencing technologies to improve the contiguity of the *P. fragariae* genome through the assembly of a greater amount of repeat rich sequence. Though this assembly still fell short of the estimated chromosome number of 10 – 12 (Brasier et al., 1999), at 180 contigs, it is a significant improvement over the previous assemblies that utilised solely short read technologies and produced relatively fragmented assemblies, comprised of >1,000 contigs each (Gao et al., 2015; Tabima et al., 2017). The increase in size of the assembly presented (91 Mb), suggests that the assembly includes an increased amount of repetitive sequence, indicating that it represents a more complete genome assembly than previous attempts. Although the assembly presented here was larger than previously reported assemblies, it is similar in size to the related Clade 7b species *P. sojae* (95 Mb; Tyler et al., 2006). This study produced assemblies of an additional ten isolates of *P. fragariae* and three isolates of the closely related raspberry pathogen *P. rubi* with short read technology. These assemblies were of similar sizes and contiguity to those previously published (Gao et al., 2015; Tabima et al., 2017).

All assemblies produced from PacBio and Illumina sequencing were annotated and putative effector genes were predicted. The gene model totals are likely inflated, due to the use of a greedy approach to effector gene prediction, to ensure the capture of all possible virulence genes. ApoplastP appeared to over-predict effectors, likely due to statistical issues arising from the use of a training set far smaller than the query set used in this study (Pritchard and Broadhurst, 2014). Interestingly, twice as many CRNs were predicted in *P. rubi* than *P. fragariae* on average (average of 118 in *P. rubi* and 67 in *P. fragariae*), though this was not consistent for all *P. rubi* isolates. In comparison to *P. sojae*, the BC-16 isolate was predicted to possess approximately 50% more RxLRs and twice as many CRNs from RNA-Seq guided gene models (Tyler et al., 2006). This difference may have been due to improvements in prediction strategies, as the number of CRNs was similar to those predicted for the Clade 1 species *P. cactorum* (Armitage et al., 2018). However for RxLR effectors this difference is likely explained by the greedy approach taken for gene prediction in this study. Following the validation of gene models by RNA-Seq data, the likely overprediction of effector genes was also shown by the low percentage of these classes of genes showing evidence of expression.

This work also allowed the resolution of *P. fragariae* race schemes between different countries, Canadian race 1 is equivalent to United Kingdom race 1, Canadian race 2 is equivalent to United Kingdom race 3 and both Canadian race 3 and United States race 4 is equivalent to United Kingdom race 2. This provided further support for the proposed gene-for-gene model of resistance in this pathosystem (van de Weg, 1997a). However, construction of orthology groups for all isolates of *P. fragariae* and *P. rubi* did not show the presence of proteins unique to the races UK1, UK2, and UK3. Additionally, the identification of variant sites, with the BC-16 genome acting as a reference, indicated there were only private variants present in UK2, with none identified in UK1 or UK3. None of these variants were in proximity to genes thought to be involved in pathogenicity. Additionally, analysis of population structure confirmed the previously described species separation of *P. fragariae* and *P. rubi* (Man in ’t Veld, 2007; Tabima et al., 2018) and identified a subpopulation consisting of the isolates of *P. fragariae* of UK1-2-3 on which further investigations focused.

Transcriptome analyses led to the identification of a strong candidate for *PfAvr2*, which was shown to be highly expressed in all *in planta* BC-16 timepoints, compared to evidence of no, or very low levels of expression in any BC-1 (UK1) or NOV-9 (UK3) samples. The strongest candidate for *PfAvr3* was also shown to be differentially expressed between races, but not as highly expressed in NOV-9 as observed for putative *PfAvr2*. RT-qPCR confirmed the *PfAvr2* results and additionally showed expression of *PfAvr2* in the other sequenced UK2 isolate, A4. The assay showed high levels of variability, which was due to variation between biological replicates, likely the result of the inability of the inoculation method to control the quantity of zoospores inoculating an individual plant. Further *in planta* experiments and RT-qPCR of additional isolates are required to investigate the observations of putative *PfAvr3*.

We propose that silencing of putative *PfAvr2* in races UK1 and UK3, enables those isolates to evade recognition in *Rpf2* possessing plants, such as “Redgauntlet”, but not in plants possessing “*Rpf1*” or “*Rpf3*”, respectively. Silencing of putative *PfAvr3* also enables races UK1 and UK2 to evade recognition in *Rpf3* possessing plants, as long as they also do not possess *Rpf1* and *Rpf3*. Whilst the exact mechanism of putative *PfAvr2* and *PfAvr3* silencing was not identified in this study, long read sequencing of a race UK3 isolate (NOV-9) identified a single SNP, 14 kb downstream of the stop codon of putative *PfAvr2* and variation in expression of transcription factors was observed in the RNA-Seq data. This indicated that epigenetic modifications could explain the observed transcriptional variation in these epialleles. Multiple examples of gain in virulence through silenced epialleles have been reported in *P. sojae*; *Avr1a*, *Avr1b*, *Avr1c*, and *Avr3a/5* (Shan et al., 2004; Qutob et al., 2009, 2013; Dong et al., 2011). In the *P. sojae* Avr3a gene, (small) sRNAs (24–26 nt in length) were identified as having a role in the heritable silencing of the gene (Qutob et al., 2013). In other isolates of *P. sojae*, variations in the promoter regions were attributed to the transcriptional differences (Dong et al., 2011). More recently, investigations of the EC-1 clonal lineage of *P. infestans* also revealed in the absence of genetic mutations, that differences in the expression level of *Avrvnt1* were detected that correlated with virulence on potato plants possessing the *Rpi-vnt1.1* gene (Pais et al., 2018). Further demonstrating that transcriptional silencing of effectors is a known mechanism that *Phytophthora* spp. employ to rapidly evade the activation of host *R*-gene-mediated immunity. However, the mechanisms underlying the rapid, adaptable and reversible silencing are poorly understood.

Adenine N6-methylation (6 mA) has been identified as an important epigenetic mark for the regulation of gene expression in *Phytophthora* spp., recent work in *P. infestans* and *P. sojae* identified evidence of 6mA methylation, alongside a lack of evidence of 5-methylcytosine (5mC) DNA methylation (Chen et al., 2018). It is also possible that chromatin modifications may be involved in controlling the expression differences, as demonstrated in *P. infestans* (van West et al., 2008; Chen et al., 2018). Investigation of these possibilities, while not achievable in the current study is a clear direction for future research, having implications in the understanding of the evolution of virulence in *Phytophthora* spp.

Nearly all *P. fragariae* isolates investigated in this study were collected from around Canada between 2001–2012. The SCRP245 isolate was identified as an intermediate between the UK1-2-3 population and the population represented by the BC-23 and ONT-3 isolates. SCRP245 could represent a rare hybrid of the two populations. However, due to the small sample size of isolates, the low number of SNP sites available for this analysis and the fact it was isolated in 1945 in the UK, at least 55 years before the other isolates, it appears more likely that SCRP245 represents a separate population but the data available were unable to resolve this fully. Further race typing with differential strawberry genotypes is required to ascertain the relationship between isolates of Canadian races 4 and 5.

Races of asexual species have been shown to evolve by the stepwise accumulation of mutations (Del Mar Jiménez-Gasco et al., 2004). One such example is the successive evolution of multiple pathotypes in a single clonal lineage of the wheat pathogen *Puccinia striiformis* f. sp. *tritici* in Australia and New Zealand (Steele et al., 2001). In comparison, our data do not indicate that *P. fragariae* has undergone simple stepwise evolution of effectors, but we rather postulate that some lineages of *P. fragariae* have been present for long periods of time in nature, evident by the large number of SNP differences and well supported branches and that the emergence of races (e.g., UK1-2-3) is fairly recent, possibly as a result of the *R*-genes deployed in commercial strawberries. The increased selection pressure on *P. fragariae* to overcome these genes, or the break-up of “wild” *R*-gene stacks upon hybridisation of octoploid strawberry species, may have led to very rapid evolution of races, in this case through epigenetic silencing of gene expression, to evade the *R*-genes present in common cultivars. Substantial further sampling from multiple geographic regions would be required to fully decipher population structure in the lineages of *P. fragariae* and the resistance status of wild octoploid *Fragaria* species. We predict that this would lead to the observation of other geographically distinct lineages of genetically similar individuals of *P. fragariae* but with similar differences in pathogenicity on strawberry. The implications of these findings highlight the potential adaptability of *P. fragariae* to modify effector expression to evade host resistance and the threat of the emergence of new races. Future strawberry breeding efforts must deploy cultivars with multiple resistance genes to mitigate against the rapid adaptation of *P. fragariae*. Identifying resistance genes that recognise the conserved core RxLRs identified in this study would enable broad-spectrum resistance to this pathogen to be deployed that would be effective against multiple races. One of the conserved highly expressed RxLRs was shown to have homology to *P. sojae Avr1b* and so identifying homologous *R*-genes to *Rsp1b* in strawberry could be a future avenue of work to provide resistance against multiple races.

## Conclusion

In conclusion, we have shown for the first time that within a distinct subpopulation of *P. fragariae* isolates, displaying remarkably low levels of polymorphisms, the ability to cause disease on a range of differing strawberry cultivars was associated with variation in transcriptional levels rather than being due to sequence variation, similar to reports in *P. sojae* and *P. infestans*. This study presents a large amount of data, including an improved, long read assembly of *P. fragariae* alongside a collection of resequenced isolates of *P. fragariae* and *P. rubi*, and transcriptome data from multiple isolates that is a valuable resource for future studies.

## Data Availability Statement

The datasets, including all assemblies and annotations, generated for this study are available on NCBI GenBank as part of BioProjects PRJNA396163 and PRJNA488213 with accession numbers of SAMN07449679–SAMN07449692. Raw sequencing reads have been deposited in the NCBI SRA, DNA-Seq reads are available with accession codes SRR7668085–SRR7668100 and *P. fragariae* RNA-Seq reads are available with accession codes SRR7764607–SRR7764615. *P. rubi* RNA-Seq reads are available with the accession codes SRR10207404–SRR10207405.

## Author Contributions

RH, CN, and TA devised the study. RH, CN, and JD co-supervised TA’s Ph.D. study, within which some of this work was undertaken. TA performed the experimental work, with contributions from CN and drafted the manuscript with input from CN, JD, and RH. AA assisted with the development of genome assembly, annotation, and orthology pipelines. MS developed elements of the variant calling pipeline and structure analysis with TA. HB performed Nanopore and MiSeq sequencing. JT, BK, BT, and NG provided RNA-Seq reads of *P*. *rubi* for genome annotation. All authors read and approved the submission.

## Conflict of Interest

The authors declare that the research was conducted in the absence of any commercial or financial relationships that could be construed as a potential conflict of interest.

## Supplementary Material

FIGURE S1 Agarose gel electrophoresis of RT-PCR reactions on representative samples from inoculation time course experiments on the “Hapil” cultivar of *Fragaria* × *ananassa*. **(A)** Gel of samples from an inoculation time course experiment with the BC-16 isolate of *Phytophthora fragariae*. **L:** 100 bp Plus Ladder (Thermo Fisher Scientific, Waltham, MA, United States). **1:** Mock inoculated *Fragaria* × *ananassa* cultivar “Hapil” plant. **2:** Time course of inoculated plants with stream water used as the flooding solution. Time points from left to right: 24 h post-inoculation (hpi), 48 hpi, 96 hpi, 144 hpi, 192 hpi, and 240 hpi. **3:** Time course of inoculated plants with Petri’s solution used as the flooding solution. Time points from left to right: 24 hpi, 48 hpi, 96 hpi, and 144 hpi. **4:** dH_2_O control. **5:** “Flamenco” gDNA control. **6:** BC-16 gDNA control. **B:** Gel of samples from an inoculation time course experiment with the BC-1 and NOV-9 isolates of *P. fragariae*. **L:** 100 bp Plus Ladder from New England Biolabs. PCR templates: **1:** Uninoculated plant cDNA. **2:** NOV-9 12 hpi cDNA. **3:** BC-1 12 hpi cDNA. **4:** NOV- 9 24 hpi cDNA. **5:** BC-1 24 hpi cDNA. **6:** NOV-9 48 hpi cDNA. **7:** BC-1 48 hpi cDNA. **8:** NOV-9 72 hpi cDNA. **9:** BC-1 72 hpi cDNA. **10:** NOV-9 96 hpi cDNA. **11:** BC-1 96 hpi cDNA. **12:** dH_2_O control. **13:** gDNA from a *F*. × *ananassa* cultivar “Hapil” plant. **14:** gDNA from BC-16 mycelium. **15:** cDNA from BC-16 mycelium.

FIGURE S2 *PfAvr3* candidate PF009_g26276 is differentially expressed in *Phytophthora fragariae* UK-1-2-3 isolates. Quantitative reverse transcription PCR of a candidate for the avirulence gene possessed by NOV-9, but not BC-1, BC-16 and A4 (PF009_g26276.t1). Plots created by the ggplot2 R package (Wickham, 2016) in R version 3.4.3 (R Core Team, 2017).

TABLE S1 Primers used in this study. Primers supplied by IDT (Leuven, Belgium).

TABLE S2 Details of all predicted genes in *Phytophthora fragariae* UK2 isolate BC-16.

TABLE S3 Details of gene names for all orthogroups in *Phytophthora fragariae* and *Phytopthora rubi*.

TABLE S4 Details of private variant sites identified in isolates of race UK2.

TABLE S5 Details of expression of genes surrounding putative *PfAvr2* (PF003_g27513).

TABLE S6 Details of expression of genes surrounding putative *PfAvr3* (PF003_g27386/PF009_g26276).

TABLE S7 Gene names of all genes identified as putative candidate avirulence genes for *Phytophthora fragariae* UK1, UK2 and UK3. Gene names are listed with respect to the isolate for which they are described as candidate avirulence genes.
