# Supplementary material for: Genomic Investigation of the Strawberry Pathogen Phytophthora fragariae Indicates Pathogenicity Is Associated With Transcriptional Variation in Three Key Races
Source: Front Microbiol. 2020 Apr 15;11:490. doi: 10.3389/fmicb.2020.00490 (PMC7174552; doi:10.3389/fmicb.2020.00490)

SUPPLEMENTARY FIGURE S1 | Agarose gel electrophoresis of RT-PCR reactions on representative samples from inoculation time course experiments on the 'Hapil' cultivar of *Fragaria* × *ananassa*.

A

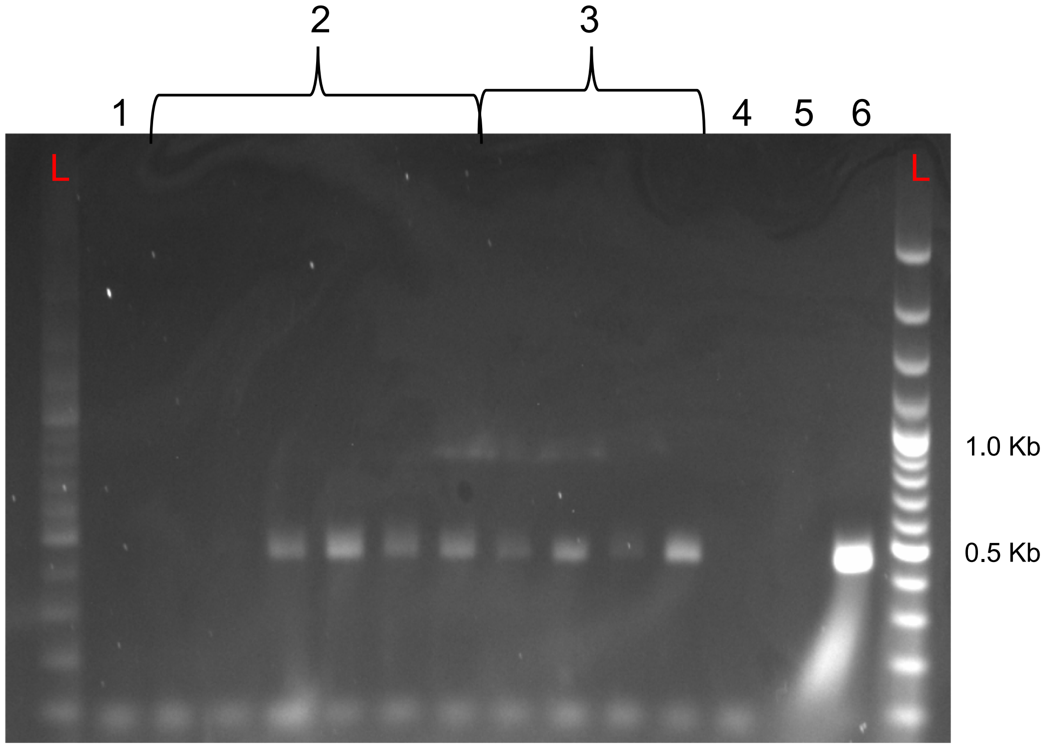

B

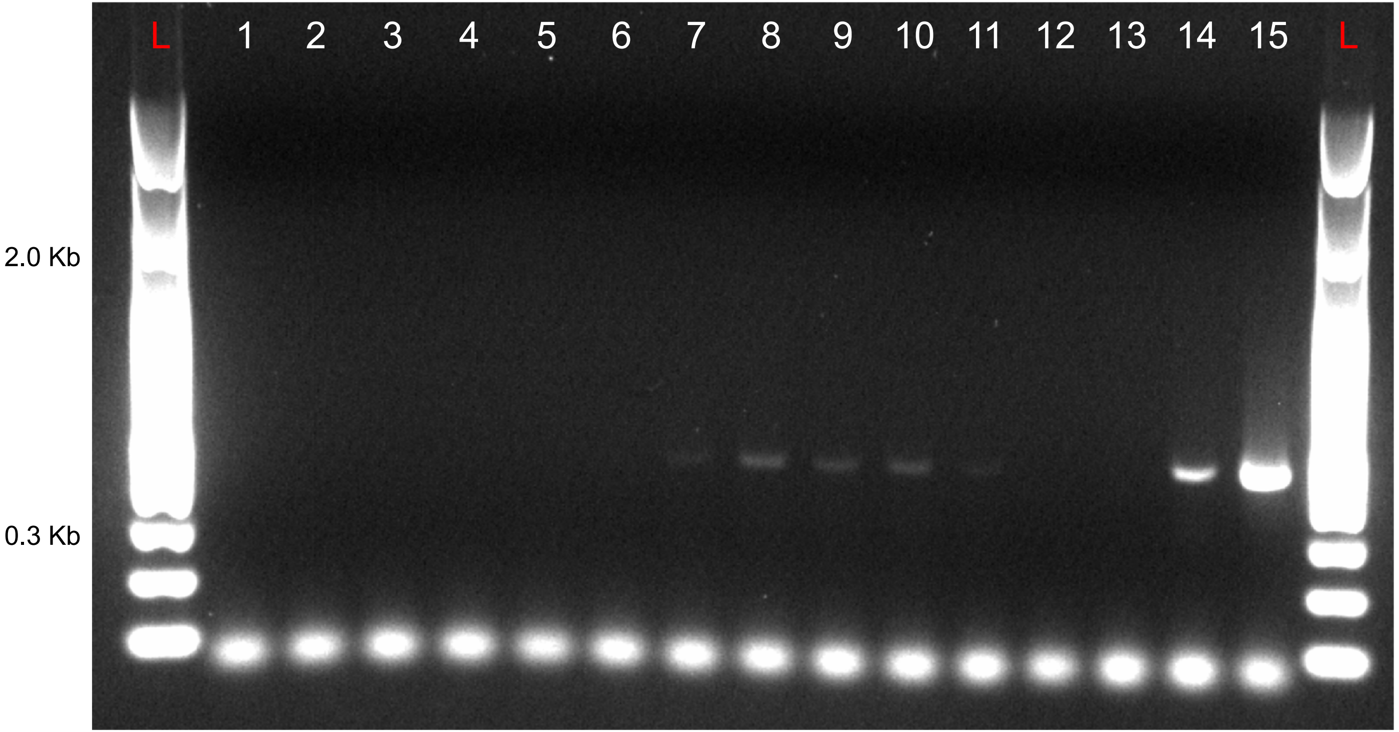

Supplement: FIGURE S1 — Agarose gel electrophoresis of RT-PCR reactions on representative samples from inoculation time course experiments on the “Hapil” cultivar of Fragaria × ananassa. (A) Gel of samples from an inoculation time course experiment with the BC-16 isolate of Phytophthora fragariae. L: 100 bp Plus Ladder (Thermo Fisher Scientific, Waltham, MA, United States). 1: Mock inoculated Fragaria × ananassa cultivar “Hapil” plant. 2: Time course of inoculated plants with stream water used as the flooding solution. Time points from left to right: 24 h post-inoculation (hpi), 48 hpi, 96 hpi, 144 hpi, 192 hpi, and 240 hpi. 3: Time course of inoculated plants with Petri’s solution used as the flooding solution. Time points from left to right: 24 hpi, 48 hpi, 96 hpi, and 144 hpi. 4: dH2O control. 5: “Flamenco” gDNA control. 6: BC-16 gDNA control. B: Gel of samples from an inoculation time course experiment with the BC-1 and NOV-9 isolates of P. fragariae. L: 100 bp Plus Ladder from New England Biolabs. PCR templates: 1: Uninoculated plant cDNA. 2: NOV-9 12 hpi cDNA. 3: BC-1 12 hpi cDNA. 4: NOV- 9 24 hpi cDNA. 5: BC-1 24 hpi cDNA. 6: NOV-9 48 hpi cDNA. 7: BC-1 48 hpi cDNA. 8: NOV-9 72 hpi cDNA. 9: BC-1 72 hpi cDNA. 10: NOV-9 96 hpi cDNA. 11: BC-1 96 hpi cDNA. 12: dH2O control. 13: gDNA from a F. × ananassa cultivar “Hapil” plant. 14: gDNA from BC-16 mycelium. 15: cDNA from BC-16 mycelium. [file Image_1.pdf]
