# Supplementary material for: Genomic Investigation of the Strawberry Pathogen Phytophthora fragariae Indicates Pathogenicity Is Associated With Transcriptional Variation in Three Key Races
Source: Front Microbiol. 2020 Apr 15;11:490. doi: 10.3389/fmicb.2020.00490 (PMC7174552; doi:10.3389/fmicb.2020.00490)

SUPPLEMENTARY FIGURE S2 | *PfAvr3* candidate PF009_g26276 is differentially expressed in *Phytophthora fragariae* UK-1-2-3 isolates.

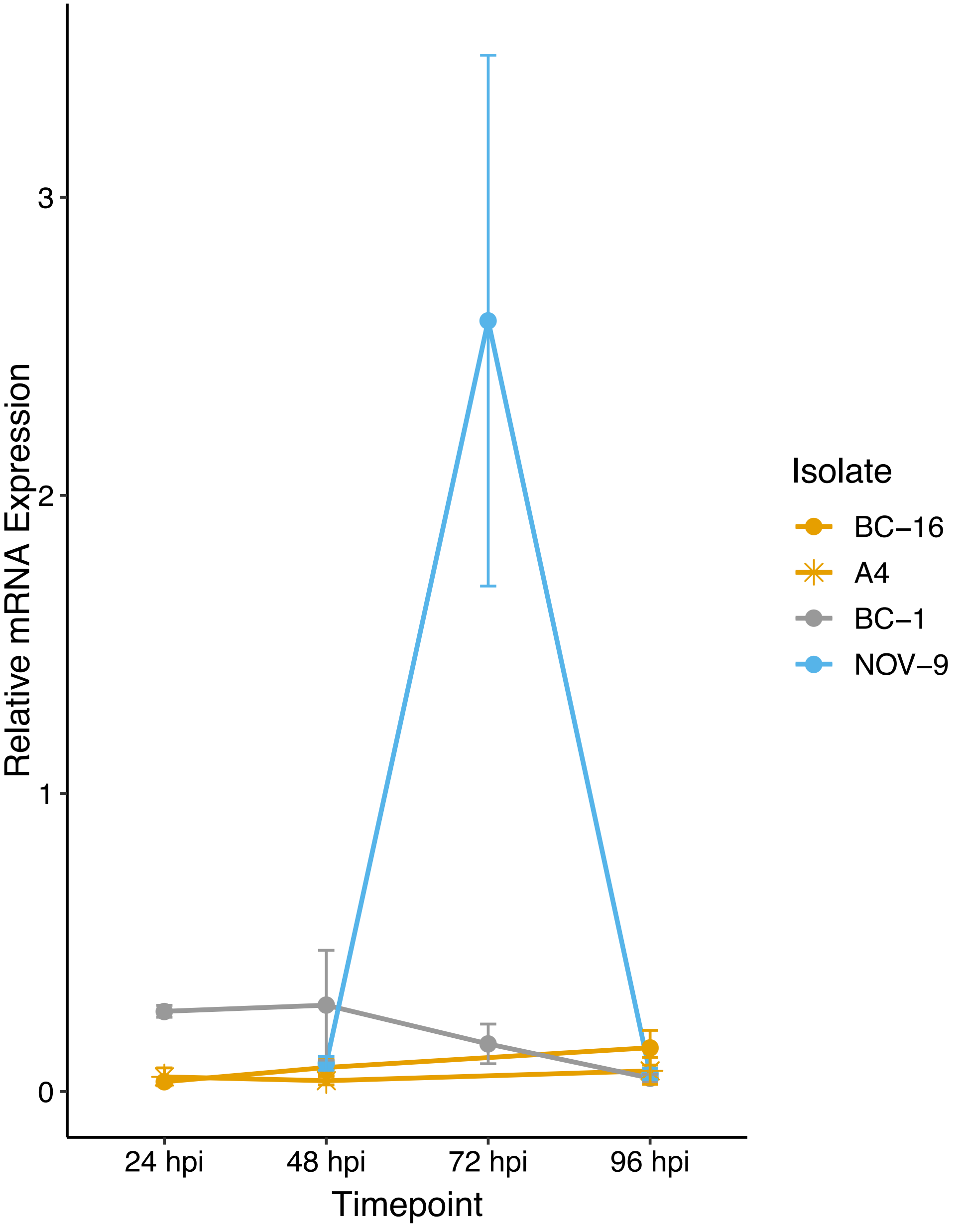

Supplement: FIGURE S2 — PfAvr3 candidate PF009_g26276 is differentially expressed in Phytophthora fragariae UK-1-2-3 isolates. Quantitative reverse transcription PCR of a candidate for the avirulence gene possessed by NOV-9, but not BC-1, BC-16 and A4 (PF009_g26276.t1). Plots created by the ggplot2 R package (Wickham, 2016) in R version 3.4.3 (R Core Team, 2017). [file Image_2.pdf]
