# Supplementary material for: Genomic Investigation of the Strawberry Pathogen Phytophthora fragariae Indicates Pathogenicity Is Associated With Transcriptional Variation in Three Key Races
Source: Front Microbiol. 2020 Apr 15;11:490. doi: 10.3389/fmicb.2020.00490 (PMC7174552; doi:10.3389/fmicb.2020.00490)
Supplement: TABLE S1 — Primers used in this study. Primers supplied by IDT (Leuven, Belgium). [file Data_Sheet_1.zip › Supplementary Table S1.DOCX]

**SUPPLEMENTARY TABLE S1 |** **Primers used in this study.** Primers supplied by IDT (Leuven, Belgium).

| **Primer Name** | **Target** | **Gene Name** | **BC-16 Gene ID** | **Primer Sequence** | **Length (bp)** | **Reference** |
| --- | --- | --- | --- | --- | --- | --- |
| Pf_Btub_F | RT-PCR | β-tubulin | PF003_g4288.t1 | 5'-GGATAACGAGGCCCTGTACG-3' | 440 | This Study |
| Pf_Btub_R |  |  |  | 5'-TGTTGTTGGGGATCCACTCG-3' |  |  |
| WS41_163F | Housekeeping | WS41 | PF003_g28439.t1 | 5'-ATCGTGCTGTACCTGGGC-3' | 156 | This Study |
| WS41_318R |  |  |  | 5'-GATCTCGCTGGGCTTGAAGG-3' |  |  |
| Btub_44F | Housekeeping | β-tubulin | PF003_g4288.t1 | 5'-CCGCGCCCGTACAGCAAC-3' | 109 | This Study |
| Btub_152R |  |  |  | 5'-TCGGAGATGACTTCCCAGAACTTG-3' |  |  |
| cAvr2_65F | Candidate Race 2 *Avr* Gene | Candidate *PfAvr2* | PF003_g27513.t1 | 5'-TGTCAAAGGCCGATCAGAGC-3' | 180 | This Study |
| cAvr2_244R |  |  |  | 5'-CGAACAAACTATCCACACCAGC-3' |  |  |
| cAvr3_F | Candidate Race 3 *Avr* Gene | Candidate *PfAvr3* | PF003_g27386.t1 | 5’-ACAAGATGGACCCGAACCTCAT-3’ | 209 | This Study |
| cAvr3_R |  |  |  | 5’-CAACCTCCTGACAGCTCCTTCAAC-3’ |  |  |
| U16SRT-F | Inter-plate calibrator | 16S | N/A | 5'-ACTCCTACGGGAGGCAGCAGT-3' | 180 | Clifford et al., 2012 |
| U16SRT-R |  |  |  | 5'-TATTACCGCGGCTGCTGGC-3' |  |  |
