# Supplementary material for: Genomic Investigation of the Strawberry Pathogen Phytophthora fragariae Indicates Pathogenicity Is Associated With Transcriptional Variation in Three Key Races
Source: Front Microbiol. 2020 Apr 15;11:490. doi: 10.3389/fmicb.2020.00490 (PMC7174552; doi:10.3389/fmicb.2020.00490)
Supplement: TABLE S1 — Primers used in this study. Primers supplied by IDT (Leuven, Belgium). [file Data_Sheet_1.zip › Supplementary Table S4.DOCX]

|  |  |  |  |  |  |  |  |  |  |  |  |
| --- | --- | --- | --- | --- | --- | --- | --- | --- | --- | --- | --- |
| **Supplementary Table S4 \| Details of all private variant sites identified in isolates of race UK2.** | | | | | | | | | | | |
| Variant ID | Race | Allele in *Phytophthora rubi* | Variant type  Reference to Alternate | Contig | Position | Region type | Upstream | | Downstream | |  |
|  |  |  |  |  |  |  | Nearest gene | Distance | Nearest gene | Distance |  |
| 1 | UK2 | Identical | G to A SNP | 14 | 964,434 | Intergenic | g11265 | 978 bp | g11266 | 3,099 bp |  |
| 2 | UK2 | Distinct | A to C SNP | 1 | 217,370 | Intergenic | g65 | 654 bp | g66 | 2,013 bp |  |
| 3 | UK2 | Distinct | T to C SNP | 2 | 1,219,725 | Intergenic | g1731 | 7,004 bp | g1732 | 3,790 bp |  |
| 4 | UK2 | Distinct | T to A SNP | 13 | 673,883 | CDS - Silent | g10482 | Within CDS | g10482 | Within CDS |  |
| 5 | UK2 | Distinct | G to A SNP | 14 | 964,434 | Intergenic | g11265 | 978 bp | g11266 | 3,099 bp |  |
| 6 | UK2 | Distinct | C to A SNP | 14 | 964,446 | Intergenic | g11265 | 990 bp | g11266 | 3,087 bp |  |
| 7 | UK2 | Distinct | A to G SNP | 14 | 1,308,196 | Intergenic | g11448 | 739 bp | g11449 | 1,375 bp |  |
| 8 | UK2 | Distinct | T to C SNP | 19 | 961,636 | CDS - Silent | g14158 | Within CDS | g14158 | Within CDS |  |
| 9 | UK2 | Distinct | Multi-Allele InDel* | 40 | 631,727 | Intergenic | g23431 | 39 bp | g23432 | 3,247 bp |  |
| 10 | UK2 | Distinct | T to C SNP | 48 | 312,824 | Intergenic | g25960 | 38 bp | g25961 | 1,997 bp |  |
| 11 | UK2 | Distinct | A to G SNP | 51 | 571,287 | CDS - L to P substitution | g27056 | Within CDS | g27056 | Within CDS |  |
| 12 | UK2 | Distinct | T to C SNP | 76 | 248,619 | Intergenic | g33391 | 4,412 bp | g33392 | 979 bp |  |
| * The reference sequence at this site in BC-16 is AC. The alternate alleles are: A, ACC, AGC and AGCC. | | | | | | | | | | |  |
