# Supplementary material for: Genomic Investigation of the Strawberry Pathogen Phytophthora fragariae Indicates Pathogenicity Is Associated With Transcriptional Variation in Three Key Races
Source: Front Microbiol. 2020 Apr 15;11:490. doi: 10.3389/fmicb.2020.00490 (PMC7174552; doi:10.3389/fmicb.2020.00490)
Supplement: TABLE S1 — Primers used in this study. Primers supplied by IDT (Leuven, Belgium). [file Data_Sheet_1.zip › Supplementary Table S5.DOCX]

**SUPPLEMENTARY TABLE S5 |** **Details of expression of genes surrounding putative *PfAvr2* (PF003_g27513).**

|  | **Orthogroup** | **BC-16 gene ID** | **Fragments Per Kilobase of transcript per Million mapped reads (FPKM)** | | | | | | | |
| --- | --- | --- | --- | --- | --- | --- | --- | --- | --- | --- |
|  |  |  | **BC-1** | | **BC-16** | | | | **NOV-9** | |
|  |  |  | **Mycelium** | **48 hpi** | **Mycelium** | **24 hpi** | **48 hpi** | **96 hpi** | **Mycelium** | **72 hpi** |
|  | OG0003811 | PF003_g27515 | 0 | 0 | 0 | 0 | 0 | 0 | 0 | 0 |
|  | OG0000375 | PF003_g27514 | 476 | 546 | 11 | 248 | 260 | 206 | 58 | 80 |
| **Putative *PfAvr2*** | **OG0026610** | **PF003_g27513** | **0** | **0** | **1** | **9,392** | **6,263** | **3,075** | **0** | **33** |
|  | OG0000960 | PF003_g27512 | 3 | 1 | 0 | 0 | 0 | 1 | 2 | 2 |
|  | OG0036362 | PF003_g27511 | 0 | 0 | 0 | 0 | 0 | 0 | -- | -- |
