# Supplementary material for: Genomic Investigation of the Strawberry Pathogen Phytophthora fragariae Indicates Pathogenicity Is Associated With Transcriptional Variation in Three Key Races
Source: Front Microbiol. 2020 Apr 15;11:490. doi: 10.3389/fmicb.2020.00490 (PMC7174552; doi:10.3389/fmicb.2020.00490)
Supplement: TABLE S1 — Primers used in this study. Primers supplied by IDT (Leuven, Belgium). [file Data_Sheet_1.zip › Supplementary Table S6.DOCX]

**SUPPLEMENTARY TABLE S6 |** **Details of expression of genes surrounding putative *PfAvr3* (PF003_g27386/PF009_g26276).**

|  | **Orthogroup** | **BC-16 gene ID** | **Fragments Per Kilobase of transcript per Million mapped reads (FPKM)** | | | | | | | |
| --- | --- | --- | --- | --- | --- | --- | --- | --- | --- | --- |
|  |  |  | **BC-1** | | **BC-16** | | | | **NOV-9** | |
|  |  |  | **Mycelium** | **48 hpi** | **Mycelium** | **24 hpi** | **48 hpi** | **96 hpi** | **Mycelium** | **72 hpi** |
|  | OG0002229 | PF003_g27388 | 0 | 0 | 0 | 0 | 0 | 0 | 0 | 0 |
|  | OG0003130 | PF003_g27387 | 0 | 0 | 0 | 0 | 0 | 0 | 0 | 0 |
| **Putative *PfAvr3*** | **OG0018589** | **PF003_g27386** | **17** | **6** | **10** | **12** | **4** | **4** | **16** | **199** |
|  | OG0018588 | PF003_g27385 | 38 | 33 | 26 | 36 | 64 | 78 | 29 | 61 |
|  | OG0018587 | PF003_g27384 | 0 | 0 | 0 | 0 | 0 | 0 | 1 | 1 |
